# Spatial metabolomics as a new avenue in plant developmental biology: insights into serine biosynthesis during spermatogenesis in ***Marchantia polymorpha***

**DOI:** 10.1080/15592324.2025.2571669

**Published:** 2025-10-17

**Authors:** Hiromitsu Tabeta, Mai Uzaki, Masami Yokota Hirai

**Affiliations:** a RIKEN Center for Sustainable Resource Science, Yokohama, Japan; b Department of Applied Biosciences, Graduate School of Bioagricultural Science, Nagoya University, Nagoya, Japan

**Keywords:** Plant morphogenesis, functional amino acids, serine biosynthesis, metabolomics, marchantia polymorpha

## Abstract

Plant development is a complex process governed by genetic regulatory networks in which metabolites play essential roles by modulating gene expression and cellular processes. While the functional importance of metabolites in plant development is increasingly recognized, their precise spatial and temporal accumulation patterns, which are closely tied to their mechanistic roles, remain poorly understood. This study highlights the need for high-resolution analyses finely tuned to specific developmental processes within the framework of plant developmental metabolomics. Using a *Marchantia polymorpha* mutant lacking 3-phosphoglycerate dehydrogenase (PGDH), an essential enzyme in serine biosynthesis and sperm formation, we demonstrated the importance of spatiotemporal metabolomics analysis. Conventional whole-organ metabolomics analysis failed to capture the difference between wild-type and mutant plants. Despite its limited resolution, however, spatial metabolomics analysis detected local metabolic changes caused by the mutation. Our results highlight the necessity of focusing on local metabolic alterations to better understand the influence of metabolism on plant development. This study illustrated how high-resolution spatial metabolomics analysis can provide new insights into the metabolic processes underlying plant development. Our findings highlight the need to refine metabolomics tools to better capture the spatial and temporal dynamics of metabolism during plant development, with broad implications for plant biology.

## Introduction

Plants possess specialized organs, such as the meristem apex, leaves, stems, and roots, all of which play essential roles in growth and survival. These organs are established and maintained through dynamic and coordinated cellular processes involving both cell proliferation and expansion. An understanding of the mechanisms that sustain these developmental systems is essential to determine how plants grow and adapt to environmental stresses. Traditionally, gene regulatory networks have been regarded as the primary drivers of plant development.^1^^,^^2^ However, there is accumulating evidence for the critical role of metabolites in modulating developmental processes. In the vegetative growth phase, *γ*-aminobutyric acid (GABA) and arginine contribute to leaf development,^3^^,^^4^ while in reproductive phases, polyamines and serine play key roles in proper floral and gametophytic development.^5‐10^ Although several primary metabolites, particularly amino acids and organic acids, are increasingly recognized as key regulators of plant development,^11^ their precise spatial and temporal accumulation patterns, which are closely linked to their mechanistic roles, remain poorly understood.

Among such functional metabolites, serine is a particularly important amino acid. In plants, serine is synthesized via three distinct metabolic pathways: the phosphorylated pathway in plastids, the glycerate pathway in the cytosol, and the glycolate pathway in mitochondria.^12^ The phosphorylated pathway of serine biosynthesis (PPSB) is initiated by dehydrogenation of 3-phosphoglycerate catalyzed by 3-phosphoglycerate dehydrogenase (PGDH). Loss-of-function mutations in the *PGDH* genes have been shown to lead to severe developmental phenotypes, including defective pollen development in *Arabidopsis thaliana* and abnormal spermatogenesis in *Marchantia polymorpha.*^6^^,^^10^ The severe phenotypes observed across phylogenetically distant plant species from the liverwort to dicots indicates a conserved and indispensable role of serine provided by PPSB in plant growth. While plants often compartmentalize the biosynthesis of bioactive metabolites to avoid interference with vital processes,^13^ the severity of PPSB disruption, despite the presence of two alternative pathways, suggests that serine biosynthesis is also tightly regulated in a spatiotemporally restricted manner.

Conventionally, metabolomics analyzes have been performed at organ-level spatial resolution, potentially leading to failure to capture the subtle and localized metabolic changes that underpin developmental transitions. To understand the functions of metabolites in specific developmental processes such as cell differentiation and organogenesis, a novel metabolomics approach with sufficiently high spatiotemporal resolution is necessary. We proposed the concept of plant developmental metabolomics,^11^ a framework that emphasizes not only the abundance of metabolites but also their precise spatial and temporal distributions within specific developmental contexts. This high-resolution view is essential to uncover previously overlooked metabolites and their roles.

Here, we applied this approach to *M. polymorpha PGDH* knockout mutant (Mp*pgdh*) and provided new insights into the role of PPSB in sperm formation. This study demonstrated that context-aware metabolomics analysis can uncover key metabolic alterations tightly linked to specific developmental outcomes and highlighted the importance of refining metabolomics tools to achieve higher spatial resolution, enabling a more precise understanding of the metabolic basis of plant development.

## Materials and methods

### Plant material and growth conditions

The male wild-type strain Takaragaike-1 (Tak-1) and male Mp*pgdh* mutant, characterized previously (Mp*pgdh*-*1*;^10^ were cultivated on half-strength Gamborg's B5 medium (pH = 5.5–5.6) containing 1% agar at 22 °C under a 16-h light/8-h dark photoperiod with fluorescent light in air (ambient CO_2_). To induce reproductive branches, the plants were grown on rockwool and irradiated with additional far-red light.

### Histological sections

Antheridial receptacles (stage 4) were dissected and fixed overnight at room temperature in FAA solution (4% formalin, 5% acetic acid, and 50% ethanol). Specimens were dehydrated by passage through an ethanol series, embedded in Technovit resin (Kulzer, Hanau, Germany) in accordance with the manufacturer's instructions, and cut into sections 6 μm thick with a microtome (RM2125 RTS; Leica Microsystems, Wetzlar, Germany), as described previously.^14^

### Widely targeted metabolomics analysis using gas chromatography–tandem quadrupole mass spectrometry

Samples of 4 mg of freeze-dried tissue powder were mixed with 1 mL of extraction solvent (80% methanol and 0.1% formic acid) in 2-mL tubes, each containing a 3-mm zirconia bead, and disrupted on a Shake Master Neo homogenizer (BMS, Tokyo, Japan). After centrifugation at 1000 × *g* at 22 °C for 1 min, aliquots of 200 μL of supernatant were transferred to 1.5-mL tubes containing 200 μL of extraction solvent and 20 μL of adonitol (0.2 mg/mL). After trimethylsilyl (TMS) derivatization as described previously,^15^ 50-μL aliquots of the supernatant were collected and analyzed by gas chromatography–tandem quadrupole mass spectrometry (GC-QqQ-MS/MS) (AOC−5000 Plus with GCMS-TQ8040; Shimadzu, Kyoto, Japan). All samples were analyzed in random order. Two quality control (QC) samples were injected at regular intervals (every six test samples) throughout the analytical run for continuous recalibration. Raw data were collected using GCMS solution software (Shimadzu). Finally, quality-filtered metabolites were selected with a signal-to-noise ratio >3 and QC relative standard deviation <30%. The GC-MS/MS parameters and multiple reaction monitoring (MRM) transitions used in the analysis were as described previously.^15^^,^^16^

## Results

**Figure 1.**
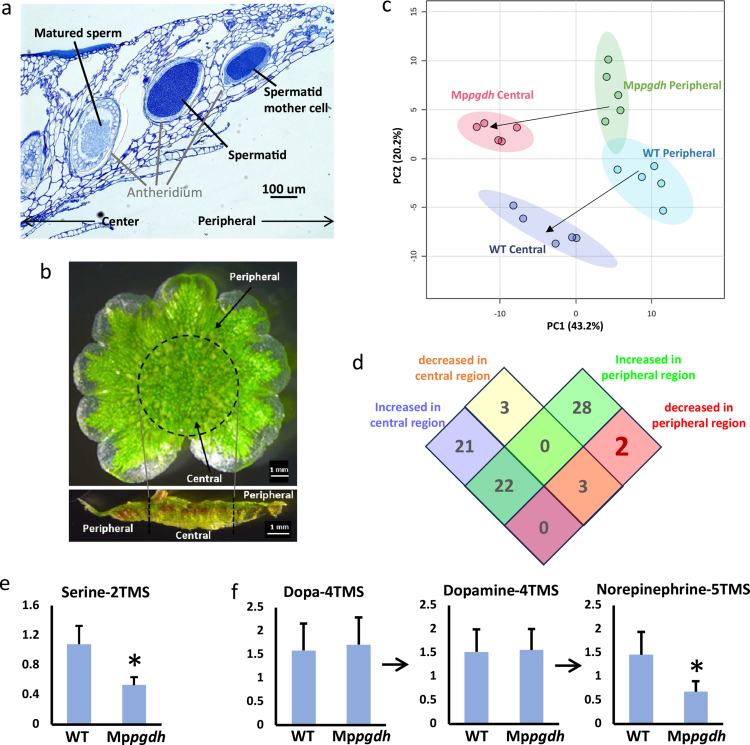
Spatial metabolomics analysis of antheridial receptacles of *M. polymorpha*. a. Histological cross-section of an antheridial receptacle from a wild-type (Tak−1) stage 4 antheridiophore. The staging criteria were based on the report by Higo et al. ^28^ Scale bar = 100 μm. b. Antheridial receptacle viewed from above (top) and in cross-section (bottom). The central and peripheral regions were separated using a 5-mm-diameter leaf punch, as indicated by the dotted circle. c. PCA score plot of metabolomics data (*n* = 5). d. Venn diagrams showing the numbers of differentially accumulated metabolites between wild-type and mutant. The differentially accumulated metabolites were identified using Student's *t* test (*n* = 6, *p *< 0.05) in combination with a fold-change (FC) threshold of >2. e. Relative serine contents in the peripheral regions. Data represent the mean + SD relative to the quality control samples (*n* = 5). Asterisks indicate significant differences compared to the wild-type (*p *< 0.05, Student's *t* test). The hydroxy and carboxyl groups in serine were derivatized with TMS for GC-MS/MS analysis. g. Relative contents of dopa, dopamine, and norepinephrine in the peripheral regions. Data represent the mean + SD relative to the quality control samples (*n* = 5). Asterisks indicate significant differences compared to wild-type (*p *< 0.05, Student's *t* test). The hydroxy group in each metabolite was derivatized with TMS. Norepinephrine is biosynthesized from tyrosine via dopa and dopamine, as indicated by the arrows.

Our previous study attributed the defect in sperm formation observed in the Mp*pgdh* mutants to the lack of serine biosynthesis via the phosphorylated pathway.^10^ However, organ-level metabolic profiling of whole antheridial receptacles failed to detect a significant reduction in serine level, highlighting a discrepancy between the metabolic profile and the developmental defect. In this study, we performed metabolic profiling with higher spatial resolution to better understand the relations between metabolic changes and developmental outcomes. In *M. polymorpha*, the antheridial receptacle of antheridiophores (male reproductive branches) presents a unique developmental gradient: the antheridia are arranged in order of developmental stage, with the youngest near the margin becoming progressively older nearer the center of the receptacle.^17^ Therefore, antheridia in the central region contain mature sperm, while those in the peripheral regions consist of spermatids and spermatid mother cells (Figure 1a). In the Mp*pgdh* mutant, spermatogenesis is arrested before specific cell division to form spermatids, and almost no sperm is generated.^10^ Notably, the internal developmental sequence and spatial arrangement of spermatids and spermatid mother cells remained comparable to those in the wild-type;^10^ Supplementary Figure 1). Leveraging this organization, we dissected the antheridial receptacle into central and peripheral regions (Figure 1b) and conducted targeted metabolomics analysis using GC-QqQ-MS/MS, focusing on primary metabolites. This spatial metabolomics approach enabled the identification of region-specific metabolic dynamics (Supplementary Figure 2), which were not detectable in previous whole-organ analyzes.^10^

On hierarchical clustering analysis (HCA), the metabolites detected in this analysis could be categorized into six clusters based on their accumulation pattern, indicating that primary metabolites were not uniformly distributed spatially in the wild-type and the mutant (Supplementary Figure 2). Interestingly, several metabolites in the C5 cluster, including cadaverine, uridine, and taurine, were reduced in Mp*pgdh* mutants (Supplementary Figure 2). These metabolites represent some intersections within plant amino acid metabolism: cadaverine originates from lysine decarboxylation;^18^ uridine links glutamine and aspartate fluxes to nucleotide-sugar biosynthesis;^19^^,^^20^ and taurine is derived from sulfur-containing amino acid pathways.^21^ So, our analysis suggests that the disruption of the PPSB pathway may affect amino acid homeostasis, thereby altering the metabolic networks associated with these compounds.

Principal component analysis (PCA) (Figure 1c) indicated that metabolic profiles differed markedly between the central and peripheral regions, revealing a metabolic gradient in the antheridial receptacle that corresponded to the developmental gradient. In the score plot (Figure 1c), the PC1 axis seemed to correspond to developmental progression (indicated by arrows), while metabolic differences between genotypes were captured along the PC2 axis. Notably, the metabolic difference between the Mp*pgdh* mutant and the wild-type was more pronounced in the central region than the peripheral region. This was likely because the developmental stages of spermatogenesis differed more substantially between the two genotypes in the central region, where mature sperm cells were formed only in the wild-type. In contrast, no visible developmental differences were observed between the genotypes in the peripheral region where spermatid mother cells were present. Therefore, the distinct metabolic profiles observed in the peripheral regions of the wild-type and the mutant (Figure 1c) may reflect metabolic characteristics that precede the appearance of visible phenotypes in the mutant. From the perspective of the plant developmental metabolomics framework, we focused on the metabolites that showed specific changes in accumulation in the peripheral region. We conducted a statistical analysis to identify differentially accumulated metabolites between the wild-type and mutant (*p *< 0.05 on Student *t*-test and fold change >2; Supplemental Table 1), which directly detected statistically significant differences and presented the results using a Venn diagram (Figure 1d). This region-specific analysis revealed that only two metabolites, serine and norepinephrine, were significantly reduced in the peripheral region of the Mp*pgdh* mutant, both showing decreases of more than twofold (Figure 1e and f).

Although our previous whole-organ analysis could not detect reductions in serine levels in the antheridial receptacle,^10^ this spatially resolved analysis revealed a significant decrease specifically in the peripheral region of the antheridial receptacle in the Mp*pgdh* mutant. In contrast, serine levels in the central region were statistically comparable between the Mp*pgdh* and wild-type. It should be noted that metabolic profiles result from a mutual causal relationship between development and metabolism. In this case, the lack of PPSB interfered with sperm formation, and, in turn, developmental defects may have perturbed metabolism. The results also suggested that serine metabolism is spatially compartmentalized within the antheridial receptacle. The inability of the central region to compensate for serine deficiency in the peripheral region suggested limited intercellular transport, implying that serine levels are regulated in a cell-autonomous manner.

In addition, we found that norepinephrine, a metabolite with neurotransmitter-like functions, was also specifically reduced in the peripheral region of the antheridial receptacle in the Mp*pgdh* mutant, despite accumulation of its precursors, DOPA and dopamine, comparable to those in the wild-type (Figure 1g). Recent studies have shown that neurotransmitter-like compounds play roles in plant organogenesis, flowering, and environmental adaptation.^22^ Given that norepinephrine has been reported to act as a flowering inducer,^23^ it may also play fundamental roles in reproductive development in *M. polymorpha*.

## Discussion

While plants biosynthesize a myriad of metabolites, the biological functions of many of them remain poorly understood. Metabolic profiles in plants undergo drastic changes during cellular differentiation and/or dedifferentiation,^24^^,^^25^ highlighting a crucial mutual relationship between plant development and metabolism. It also remains unclear whether the “metabolic differentiation” of cells – i.e., the acquisition of cell type-specific metabolic capabilities – precedes or follows the morphological differentiation of cells.^26^ To fully understand these interrelated processes, spatially resolved information on metabolite dynamics is essential. To address this need, we introduced the concept of plant developmental metabolomics^11^ and applied it to elucidate the spatiotemporal role of PPSB in *M. polymorpha*. By analyzing metabolic profiles in the Mp*pgdh* mutant with region-specific resolution, we demonstrated that serine levels are locally regulated through the PPSB, an insight that was not captured in our previous study. Interestingly, many metabolites were increased in the Mp*pgdh* mutant (Figure 1d, Supplementary Table 1), which may reflect flux rerouting into compensatory pathways, altered C/N balance due to relief of upstream bottlenecks, and the induction of stress-responsive metabolism caused by the blockage of PPSB.

Notably, the reduction in serine levels in the mutant was observed only in the peripheral region of the antheridial receptacles, where diagonal cell divisions – characteristic of spermatid formation – had not yet occurred (Figure 1d and e). This result suggested that the PPSB may function in the peripheral region to support proper sperm development. Spermatogenesis in *M. polymorpha* depends on transcription factors such as DUO1 and KANADI, which regulate antheridium development and spatial patterning.^27,^^28^ Within this framework, serine deficiency may locally disrupt the metabolic environment necessary for these proteins to function properly. Furthermore, since serine is a building block for proteins and a precursor to lipids such as ceramide, delaying the supply of these biosynthetic precursors could impair cell structure and division in specific peripheral regions, resulting in the failure to form sperm. In addition to reduce of serine contents, norepinephrine also decreased in peripheral regions in Mp*pgdh* (Figure 1f). Norepinephrine has been detected in plants as part of the catecholamine metabolic network and may be involved in stress adaptation, antioxidant responses, and developmental regulation, possibly supporting gamete maturation in reproductive tissues.^29^ The tissue-specific reduction of norepinephrine in PPSB-deficient mutants may be due to impaired serine-derived one-carbon and redox metabolism, which in turn may influence male gamete differentiation.

However, in contrast to this finding, our previous analysis using a GUS reporter showed that Mp*PGDH* is expressed in the middle area of antheridial receptacles.^10^ To achieve a comprehensive understanding of the complex interplay between development and metabolism, more advanced metabolomics and transcriptomics tools with higher spatial and temporal resolution are required.

Mass spectrometry imaging (MSI), which combines mass spectrometry with cross-sectioned organs/tissues, offers a promising approach for spatial metabolomics and continues to advance rapidly. Improvements in the sensitivity of mass spectrometers have significantly enhanced both spatial and mass resolution, enabling metabolite localization analysis at near single-cell resolution.^30‐32^ However, despite significant advances in MSI for animal experimental systems, its application to plants remains challenging due to their unique structural and biochemical characteristics. In particular, sample preparation – especially tissue sectioning – remains a major bottleneck. As chemical fixation can lead to metabolite loss, plant tissues generally need to be sectioned without fixation, making it difficult to prepare clear sections without metabolite leakage or distortion.

The development of robust and versatile sample preparation methods will enhance the utility of MSI in plant metabolomics, enabling the identification of previously unrecognized links between metabolites and key developmental processes such as morphogenesis and cell differentiation. Advances in sample preparation techniques will not only provide a deeper understanding of known metabolites but also enable the discovery of novel regulatory metabolites that may be overlooked in conventional analyzes. However, in terms of quantitative accuracy, conventional spatial analysis based on dissecting organs into defined regions still surpasses MSI, as MSI does not provide concentration data for metabolites. Laser microdissection (LMD) can be used to improve spatial resolution. Accordingly, we propose a combined approach utilizing MSI and conventional spatial analysis with LMD to achieve comprehensive spatial metabolomics profiling. Ultimately, advances in integrative analytical techniques will open new frontiers in decoding the chemical basis of plant morphogenesis and its molecular function at cellular resolution, leading to a more comprehensive and mechanistic understanding of plant development.

## Disclousre statement

The authors report there are no competing interests to declare.

## Acknowledgments

We thank Dr. Mengyo Wang (RIKEN Center for Sustainable Resource Science) and Dr. Kinuka Ohtaka (RIKEN Center for Sustainable Resource Science, Japan Women's University) for special technical support and scientific discussion.

## Supplementary Material

Supplementary material Supplementary Table 1. Differentially accumulated metabolites selected by Fold Change.

Supplementary material Supplementary figure 1. Antheridium morphology in WT and Mppgdh mutant. Sections of the antheridium at the spermatid mother cell, spermatid, and mature stages. The spermatid mother cell appears comparable between WT and Mppgdh. However, spermatogenesis in Mppgdh was arrested before maturation. Each cell structure visible in the FE-SEM images was described in Wang et al. (2024).

Supplementary material Supplementary figure 2. Metabolic profiles of the central and peripheral regions of antheridial receptacles (stage 4). 157 quality-filtered metabolites detected by GC-QqQ-MS were categorized into six clusters (C1–C6) based on their accumulation patterns. The number of trimethylsilylated structures is represented by –nTMS. Data are shown as Z-scores of the average content relative to that of the quality control samples (n = 5).
