# Supplementary material for: Spatial metabolomics as a new avenue in plant developmental biology: insights into serine biosynthesis during spermatogenesis in Marchantia polymorpha
Source: Plant Signal Behav. 2025 Oct 17;20(1):2571669. doi: 10.1080/15592324.2025.2571669 (PMC12536615; doi:10.1080/15592324.2025.2571669)
Supplement: Supplementary material — Supplementary figure 1. Antheridium morphology in WT and Mppgdh mutant. Sections of the antheridium at the spermatid mother cell, spermatid, and mature stages. The spermatid mother cell appears comparable between WT and Mppgdh. However, spermatogenesis in Mppgdh was arrested before maturation. Each cell structure visible in the FE-SEM images was described in Wang et al. (2024). [file KPSB_A_2571669_SM2793.pdf]

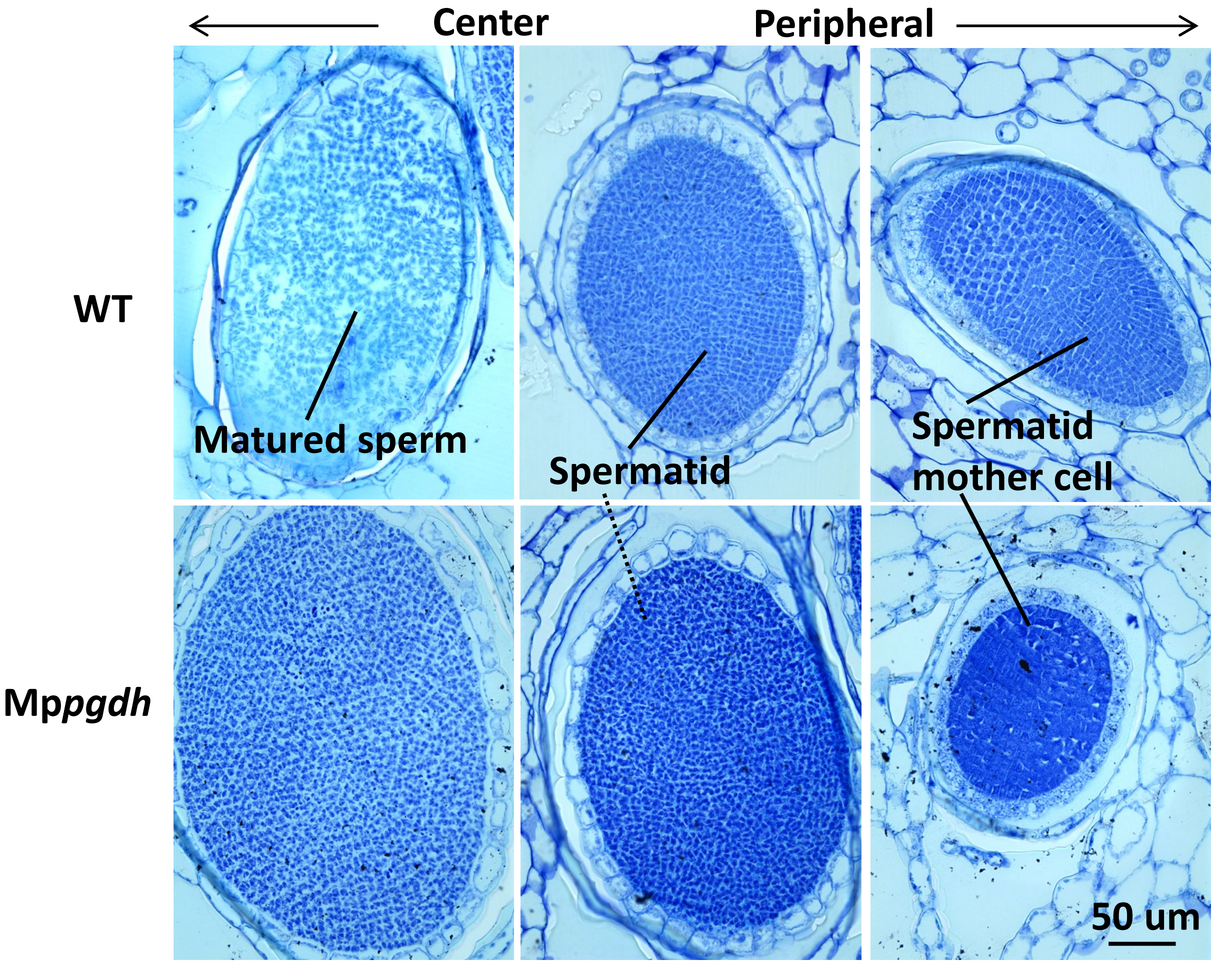

Supplementary figure1. Antheridium morphology in WT and Mp*pgdh* mutant.
Sections of the antheridium at the spermatid mother cell, spermatid, and mature stages. The spermatid mother cell appears comparable between WT and Mp*pgdh*. However, spermatogenesis in Mp*pgdh* was arrested before maturation. Each cell structure visible in the FE-SEM images was described in Wang et al. (2024).
