# Supplementary material for: Spatial metabolomics as a new avenue in plant developmental biology: insights into serine biosynthesis during spermatogenesis in Marchantia polymorpha
Source: Plant Signal Behav. 2025 Oct 17;20(1):2571669. doi: 10.1080/15592324.2025.2571669 (PMC12536615; doi:10.1080/15592324.2025.2571669)
Supplement: Supplementary material — Supplementary figure 2. Metabolic profiles of the central and peripheral regions of antheridial receptacles (stage 4). 157 quality-filtered metabolites detected by GC-QqQ-MS were categorized into six clusters (C1–C6) based on their accumulation patterns. The number of trimethylsilylated structures is represented by –nTMS. Data are shown as Z-scores of the average content relative to that of the quality control samples (n = 5). [file KPSB_A_2571669_SM2792.pdf]

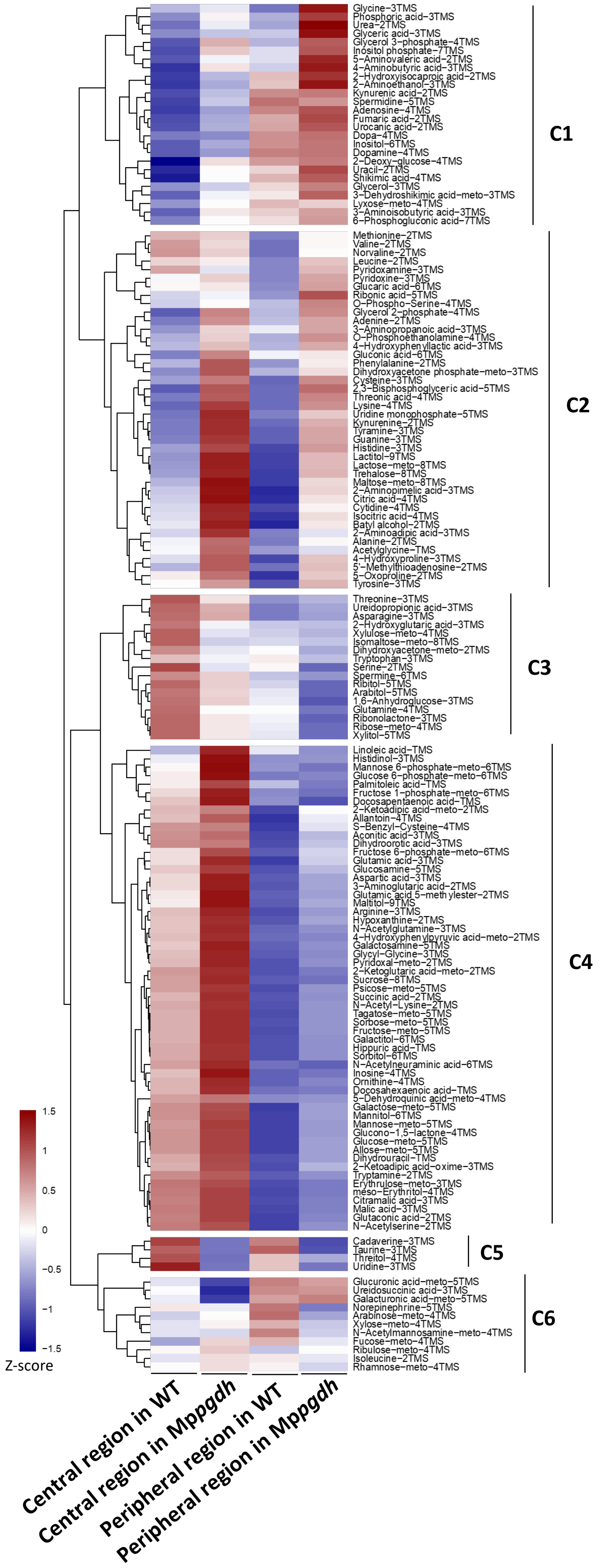

Supplementary figure 2. Metabolic profiles of the central and peripheral regions of antheridial receptacles (stage 4). 157 quality-filtered metabolites detected by GC-QqQ-MS were categorized into six clusters (C1–C6) based on their accumulation patterns. The number of trimethylsilylated structures is represented by –nTMS. Data are shown as Z-scores of the average content relative to that of the quality control samples (n = 5).
